# Targeting Cytokine-Mediated Inflammation in Brain Disorders: Developing New Treatment Strategies

**DOI:** 10.3390/ph18010104

**Published:** 2025-01-15

**Authors:** Rahul Mallick, Sanjay Basak, Premanjali Chowdhury, Prasenjit Bhowmik, Ranjit K. Das, Antara Banerjee, Sujay Paul, Surajit Pathak, Asim K. Duttaroy

**Affiliations:** 1 A.I. Virtanen Institute for Molecular Sciences, Faculty of Health Sciences, University of Eastern Finland, 70211 Kuopio, Finland; rahul.mallick@uef.fi; 2 Molecular Biology Division, ICMR-National Institute of Nutrition, Indian Council of Medical Research, Hyderabad 500007, India; sba_bioc@yahoo.com; 3 Institute of Public Health and Clinical Nutrition, School of Medicine, Faculty of Health Sciences, University of Eastern Finland, 70210 Kuopio, Finland; premacho@student.uef.fi; 4 Department of Chemistry, Uppsala Biomedical Centre, Uppsala University, SE-751 23 Uppsala, Sweden; prasenjitbhowmik200@gmail.com; 5 Department of Textile Engineering, Green University of Bangladesh, Narayanganj 1461, Bangladesh; 6 Department of Health and Biomedical Sciences, University of Texas Rio Grande Valley, Brownsville, TX 78520, USA; ranjitd37@gmail.com; 7 Faculty of Allied Health Sciences, Chettinad Hospital and Research Institute (CHRI), Chettinad Academy of Research and Education (CARE), Chennai 603103, India; antara.banerjee27@gmail.com (A.B.); surajit.pathak@gmail.com (S.P.); 8 School of Engineering and Sciences, Tecnologico de Monterrey, Queretaro 76130, Mexico; spaul@tec.mx; 9 Department of Nutrition, Institute of Basic Medical Sciences, Faculty of Medicine, University of Oslo, 0317 Oslo, Norway

**Keywords:** cytokine-mediated neuroinflammation, brain disorders therapy, pro-inflammatory cytokines, targeted drug delivery, biomarkers in neuroinflammation

## Abstract

Cytokine-mediated inflammation is increasingly recognized for playing a vital role in the pathophysiology of a wide range of brain disorders, including neurodegenerative, psychiatric, and neurodevelopmental problems. Pro-inflammatory cytokines such as interleukin-1 (IL-1), tumor necrosis factor-alpha (TNF-α), and interleukin-6 (IL-6) cause neuroinflammation, alter brain function, and accelerate disease development. Despite progress in understanding these pathways, effective medicines targeting brain inflammation are still limited. Traditional anti-inflammatory and immunomodulatory drugs are effective in peripheral inflammatory illnesses. Still, they face substantial hurdles when applied to the central nervous system (CNS), such as the blood–brain barrier (BBB) and unwanted systemic effects. This review highlights the developing treatment techniques for modifying cytokine-driven neuroinflammation, focusing on advances that selectively target critical cytokines involved in brain pathology. Novel approaches, including cytokine-specific inhibitors, antibody-based therapeutics, gene- and RNA-based interventions, and sophisticated drug delivery systems like nanoparticles, show promise with respect to lowering neuroinflammation with greater specificity and safety. Furthermore, developments in biomarker discoveries and neuroimaging techniques are improving our ability to monitor inflammatory responses, allowing for more accurate and personalized treatment regimens. Preclinical and clinical trial data demonstrate the therapeutic potential of these tailored techniques. However, significant challenges remain, such as improving delivery across the BBB and reducing off-target effects. As research advances, the creation of personalized, cytokine-centered therapeutics has the potential to alter the therapy landscape for brain illnesses, giving patients hope for better results and a higher quality of life.

## 1. Introduction

Cytokine-mediated inflammation has emerged as a critical mechanism in the pathogenesis of a variety of brain disorders, including neurodegenerative diseases, psychiatric problems, and neurodevelopmental disorders. Cytokines are essential signaling molecules in the CNS. They govern immunological responses, neuronal function, and tissue homeostasis. Under normal circumstances, cytokine production is closely controlled, producing a delicate balance of pro-inflammatory and anti-inflammatory signals. However, in certain neurological disorders, the dysregulation of cytokine signaling can lead to persistent neuroinflammation, contributing to disease initiation, progression, and poor prognosis [1,2].

Pro-inflammatory cytokines such as interleukin-1 (IL-1), tumor necrosis factor alpha (TNF-α), and interleukin-6 (IL-6) have been linked to neuroinflammation in illnesses such as Alzheimer’s disease (AD), Parkinson’s disease (PD), depression, and multiple sclerosis (MS) [3,4,5,6]. Elevated levels of cytokines such as IL-1 and TNF-α in the brain have been related to synaptic dysfunction, neurodegeneration, and behavioral changes, emphasizing their crucial role in brain health [3]. In addition to these chronic neurodegenerative disorders, acute brain lesions such as strokes and traumatic brain injuries (TBIs) cause a powerful cytokine response that exacerbates neuronal damage and hampers recovery [7,8].

Despite advances in our understanding of cytokines’ involvement in brain diseases, current approaches to treating neuroinflammation remain restricted. Traditional anti-inflammatory therapies, such as nonsteroidal anti-inflammatory medications (NSAIDs) and corticosteroids, frequently fail to penetrate the BBB and can have systemic adverse effects, limiting their clinical relevance in treating CNS illnesses [9,10]. More recent approaches have centered on developing targeted medicines that directly control cytokine signaling pathways, promising better selectivity and fewer off-target effects. These include cytokine-specific inhibitors, monoclonal antibodies, and small-molecule antagonists [11,12,13].

This article’s primary goal is to summarize the most recent advances in targeting cytokine-mediated inflammation as a treatment strategy for brain diseases. The important roles of cytokines in brain pathology, present and emerging therapeutics, and the obstacles to targeting neuroinflammation in the CNS are also presented. We also highlight emerging medication delivery technologies, such as nanotechnology and gene-based approaches, boosting the ability to modulate brain inflammation selectively. Finally, we discuss biomarkers’ potential to monitor inflammatory responses and guide treatment methods in personalized medicine techniques.

## 2. Cytokine-Mediated Inflammation in the Brain

Cytokine-mediated inflammation is an integral part of the immune response in the CNS, acting as both a protective mechanism and a potential cause of pathology. In the steady state, the CNS maintains a balanced immune milieu primarily governed by microglia, the brain’s resident immune cells, and astrocytes, with peripheral immune cells being recruited only in response to pathological conditions such as injuries or infection. Cytokines, a varied set of signaling molecules that include interleukins, chemokines, and tumor necrosis factors, play critical roles in the immunological signaling network. They perform a dual role of either boosting neuroprotection and repair or, when dysregulated, generating chronic inflammation and neurodegeneration [4,14,15,16].

Cytokines including IL-1β, TNF-α, and IL-6 activate signaling cascades including NF-κB and JAK/STAT, leading to neuroinflammation and BBB disruption [17,18,19]. Furthermore, cytokines can impair BBB integrity, enabling peripheral immune cell activity and greater cytokine inputs, thereby aggravating CNS inflammation [20,21,22]. This compromise of BBB integrity is a key factor in neuroinflammatory diseases, as it allows for the infiltration of circulating immune cells and the accumulation of additional pro-inflammatory cytokines, further amplifying the inflammatory response. As a result, a sustained inflammatory state can exacerbate neurological injuries and contribute to disease progression.

**Figure 1:**
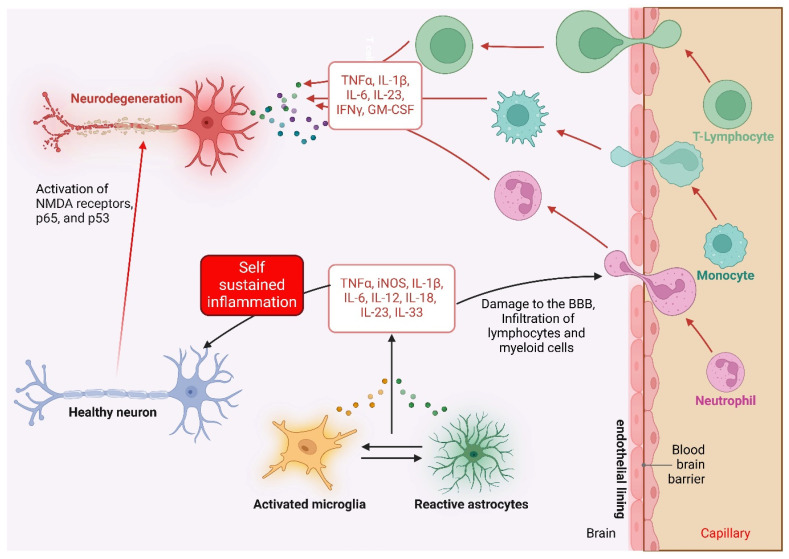
Cytokine pathways in neuroinflammation. In neurodegenerative conditions, stromal cells (e.g., astrocytes) and microglia release proinflammatory cytokines in response to homeostatic imbalances. Early cytokine release may aid repair, but chronic secretion leads to neuronal damage and loss of tissue function. In addition, leukocyte infiltration and BBB disruption contribute to neuroinflammatory conditions. Lymphocytes and myeloid cells drive inflammation through cytokines such as IL-1β and IL-6, affecting neurons. IL-23 amplifies T cell pathogenicity, while GM-CSF activates monocyte-derived cells, exacerbating tissue damage. Other key players include IFNγ and TNFα, which fuel the inflammatory cascade.

Pro-inflammatory cytokines, including IL-1β, TNF-α, and IL-6, are crucial in triggering and maintaining neuroinflammatory reactions (Figure 1). High levels of IL-1β have been associated with neuronal excitability, oxidative stress, and neuronal death in disorders such as AD, PD, and MS [23,24,25]. TNF-α, a key cytokine in neuroinflammation, impairs synaptic plasticity, causing cognitive deficits and memory impairments in neurodegenerative and psychiatric illnesses [3,26]. IL-6 has been implicated in neurogenesis and synaptic plasticity, with its dysregulation being linked to cognitive dysfunction in mental illnesses such as depression [27,28,29]. Table 1 summarizes the role of cytokines in brain disorders.

Comorbid diseases such as hypertension, diabetes, and obesity aggravate neuroinflammatory responses by disrupting vascular integrity and promoting systemic inflammation. Hypertension can activate pro-inflammatory cytokines such as IL-6 and TNF-α, leading to BBB disruption and neuronal damage in conditions such as strokes and TBIs [30]. These comorbidities exacerbate the neuroinflammatory cascade, hastening the evolution of acute and chronic CNS diseases.

Transitioning from acute to chronic neuroinflammation is critical for disease progression in many brain illnesses. For example, in AD, cytokine-driven inflammation increases amyloid-beta (Aβ) aggregation and tau hyperphosphorylation, disrupting synaptic transmission and leading to neuronal death [4,31]. Similarly, persistent cytokine activity in PD and MS contributes to ongoing neuroinflammation, resulting in neuronal loss and demyelination, respectively. The cumulative impact of chronic cytokine signaling underscores the significance of therapeutic strategies aimed at curbing pro-inflammatory cytokine activity while preserving the essential protective functions of immune signaling [27,28,29]. Understanding cytokine-mediated inflammation in the brain has provided insights highlighting the promise of targeted anti-cytokine therapy. While cytokine responses are necessary for beginning immune responses and facilitating repair, their dysregulation in chronic brain diseases emphasizes the importance of treatment techniques that can selectively control these signals without impairing general immune function. By investigating the many roles of cytokines in neuroinflammation, new strategies are being developed to attenuate their detrimental consequences, which may eventually delay or even reverse disease progression in CNS illnesses.

## 3. Brain Disorders Associated with Cytokine Dysregulation

**Figure 2:**
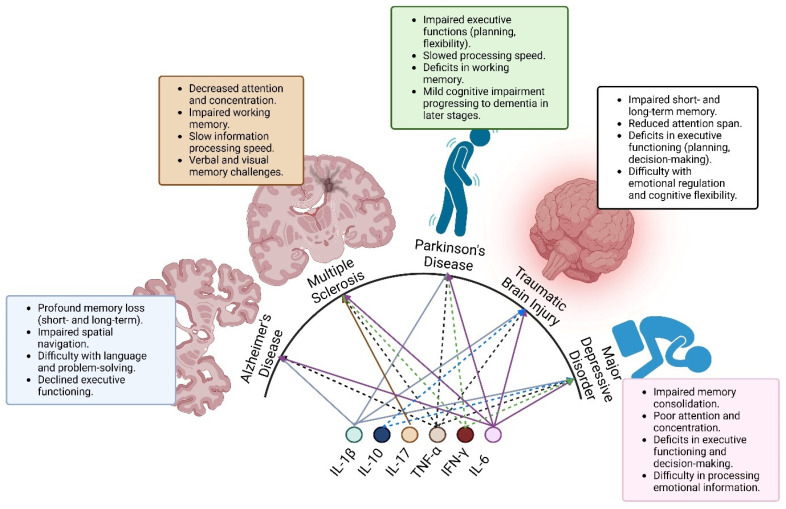
Brain disorders and cytokine dysregulation. Cytokines are closely linked to cognitive impairments in neurological disorders. Notably, IL-6 and TNF-α are common cytokines contributing to cognitive dysfunction across all disorders. Both solid and dotted lines denote cytokine involvement in the neurological disorders.

Cytokine dysregulation is a common hallmark of numerous brain disorders, including neurodegenerative, psychiatric, neurodevelopmental, autoimmune, and acute brain injury syndromes (Figure 2). Rather than being limited to specific disorders, cytokine dysregulation is a general concept of immunological imbalance that causes neuroinflammation, neurodegeneration, and circuit abnormalities. This common mechanism underpins cognitive, motor, and emotional impairment reported for various diseases [4,32,33].

Comorbidities such as hypertension, atherosclerosis, and diabetes not only predispose people to developing CNS illnesses but also exacerbate the cytokine dysregulation seen in these conditions. Hypertension has been linked to higher levels of IL-6 and TNF-α, making the CNS more susceptible to neuroinflammatory injury [30]. Obesity and diabetes have been linked to increased levels of IL-1β and resistin, which can exacerbate neuroinflammatory processes.

Cytokine dysregulation is characterized by the chronic activation of microglia, astrocytes, and peripheral immune cells, which release pro-inflammatory cytokines such as IL-1β, TNF-α, and IL-6. These cytokines begin and sustain a neuroinflammation feedback loop, culminating in BBB breakdown, increased oxidative stress, synaptic dysfunction, and neuronal death. The endurance of this inflammatory environment is a significant element in the genesis and progression of neurological disorders [23,34,35].

Cytokine dysregulation increases the aggregation of misfolded proteins in neurodegenerative diseases and compromises synaptic integrity [36]. In AD, cytokine-driven inflammation causes Aβ plaque deposition and tau hyperphosphorylation, which harm neuronal function and survival [37]. In PD, cytokines such as TNF-α contribute to the death of dopaminergic neurons, causing motor symptoms [4,11,37,38,39].

Cytokine dysregulation has an impact on neurogenesis and synaptic plasticity in neurological conditions such as major depressive disorder (MDD) and schizophrenia, especially in the hippocampus and prefrontal cortex. High levels of IL-6 and TNF-α are linked to mood and cognitive abnormalities, while abnormal cytokine signaling in schizophrenia is linked to poor brain connections and developmental alterations [40,41,42]. These findings imply that pro-inflammatory cytokines play a role in altered emotional and cognitive processing, supporting the use of immune-modulating therapy in psychiatric care [43,44].

Cytokine dysregulation is also strongly associated with neurodevelopmental disorders, including autism spectrum disorder (ASD). Evidence suggests that prenatal exposure to increased cytokines such as IL-6 can affect fetal brain development, increasing the chances of ASD development [45]. Higher levels of pro-inflammatory cytokines have been associated with aberrant synapse formation and connection in ASD patients, which may explain the social and cognitive abnormalities reported [46,47].

Acute brain injuries, such as strokes and TBIs, result in a fast cytokine response aimed at limiting damage and boosting repair. However, high or sustained cytokine activation can exacerbate injury severity. IL-1β and TNF-α breach the BBB and cause neuronal cell death in stroke, while IL-6 and IL-8 enhance oxidative stress and secondary damage in TBI [48,49,50]. Chronic inflammation after such injuries raises the possibility of developing long-term cognitive deficits and neurodegenerative diseases [51,52].

Autoimmune disorders such as MS are another example of cytokine dysfunction. In MS, cytokines such as IL-17 and IFN-γ recruit immune cells into the CNS and assault the myelin sheath, causing demyelination and neurological dysfunction [53,54,55]. The chronic inflammatory milieu in MS accelerates disease progression and cognitive deterioration, stressing the importance of cytokine-targeted treatment approaches.

The overriding principle of cytokine dysregulation in neurological disorders emphasizes the possibility for cytokine-targeted treatments. Emerging treatments are designed to lower the inflammatory load, halt disease development, and enhance clinical outcomes by targeting common immunological imbalance pathways such as BBB disintegration, synaptic dysfunction, and persistent neuroinflammation. Research into cytokine signaling pathways and their role in neuroinflammatory diseases paves the way for the development of more effective, customized treatments for a variety of CNS disorders.

## 4. The Blood–Brain Barrier (BBB) and Its Role in Cytokine-Mediated Inflammation

The BBB regulates CNS homeostasis by acting as a selective physical and biochemical barrier that prevents peripheral chemicals, immune cells, and pathogens from entering the brain parenchyma [20]. Tight connections between endothelial cells, basement membrane support, and interactions with astrocytes and pericytes all contribute to the BBB’s stability [56]. However, many CNS illnesses, particularly those with an inflammatory component, disrupt the BBB, resulting in increased permeability and infiltration of peripheral immune components [57].

Cytokine-mediated inflammation contributes significantly to BBB breakdown. Pro-inflammatory cytokines such as IL-1β, TNF-α, and IL-6 can reduce the expression of tight junction proteins (e.g., occludin and claudins) and increase the production of matrix metalloproteinases (MMPs), which damage the extracellular matrix and weaken the BBB [57]. This breakdown permits peripheral immune cells, such as lymphocytes and monocytes, to invade the CNS, causing neuroinflammation and aggravating disease pathology [58].

The degree of BBB disruption varies according to the specific CNS illness. Chronic low-level inflammation in neurodegenerative diseases such as AD and PD causes mild but persistent BBB leakage. This enables a delayed infusion of peripheral immune components, which leads to neural dysfunction and cognitive impairment [59]. In contrast, acute traumas such as strokes and TBIs produce a more severe and abrupt collapse of the BBB, resulting in a widespread influx of immune cells and pro-inflammatory cytokines [60,61]. This immediate disturbance causes oxidative stress, edema, and excitotoxicity, exacerbating brain injuries [49]. MS, an autoimmune demyelinating illness, represents another type of BBB malfunction. Driven by cytokines such as IL-17 and IFN-γ, activated immune cells cross the damaged BBB and target myelin sheaths, resulting in demyelination and neurological impairment [54,62].

The existence of a damaged BBB in certain illnesses has serious consequences for drug delivery [63]. While the BBB is commonly regarded as a barrier to CNS-targeted therapies, its collapse in disorders such as strokes, TBIs, and MS poses both obstacles and opportunities [22]. On the one hand, BBB disruption increases the permeability of therapeutic medicines, potentially improving the delivery of drugs to damaged brain regions [64]. Conversely, a loss of selectivity combined with an inflammatory milieu may result in off-target effects or neurotoxicity [65]. Thus, innovative delivery strategies must take into account the dynamic status of the BBB in CNS diseases.

Recent innovations in drug delivery technologies, such as nanoparticles and liposomes, are intended to take advantage of alterations in BBB permeability in disease states [66,67]. Nanoparticles can be engineered to penetrate the intact BBB via receptor-mediated transcytosis; however, when the BBB is broken, they can use paracellular diffusion pathways to reach the brain parenchyma [66,68]. Importantly, delivery techniques for acute and chronic CNS illnesses must be disease-specific, as the amount and duration of BBB permeability differ significantly according to the situation. For example, clinicians carrying out acute medication administration following a TBI or stroke may need to target the temporary “window of permeability” when the BBB is most damaged, but chronic illnesses such as AD may necessitate delivery systems that cross an intact but malfunctioning BBB [69,70].

Overall, while the BBB poses a considerable hurdle to CNS-targeted treatments, its collapse in inflammatory brain diseases provides an opportunity to increase drug delivery. Understanding the processes of BBB breakdown across diverse CNS disorders can aid in developing tailored treatments that capitalize on disease-specific permeability differences. Therapeutic techniques must be designed and applied in such a way that the advantages of increased medication penetration are weighed against the dangers of nonspecific targeting and potential neurotoxicity.

## 5. Current Therapeutic Approaches for Inflammation in Brain Disorders

Treatments for inflammation in brain diseases have progressed dramatically, with efforts now extending beyond typical anti-inflammatory medications to include targeted immunomodulators. Given the significance of cytokines in neuroinflammation, current therapies are designed to attenuate pro-inflammatory signaling, restore immunological balance, and alleviate the detrimental effects of chronic inflammation on neuronal function and survival. Here, we discuss the various classes of anti-inflammatory medications used to treat brain disorders and the hurdles and recent breakthroughs that show hope for improving the outcomes of patients with neuroinflammatory diseases.

### 5.1. Nonsteroidal Anti-Inflammatory Drugs (NSAIDs)

NSAIDs are commonly used because of their ability to suppress cyclooxygenase (COX) enzymes, which reduce prostaglandin production and, as a result, inflammation. Long-term usage of NSAIDs has been proven to lessen the risk of AD, probably due to their impact on neuroinflammation [71,72]. NSAIDs have been explored as neuroprotective drugs; however, their low BBB penetration and off-target effects hinder success [10,73].

### 5.2. Corticosteroids

Corticosteroids, such as prednisone and dexamethasone, are powerful anti-inflammatory medications that suppress immune responses via glucocorticoid receptor signaling. They are frequently used in autoimmune disorders and acute neuroinflammatory situations, such as MS relapses and TBIs, to reduce swelling and immune activation [74,75,76]. Chronic corticosteroid use, however, is associated with a variety of adverse effects, including immunosuppression, osteoporosis, and metabolic abnormalities, limiting the effectiveness of these drugs in long-term therapy for neurodegenerative illnesses. Furthermore, corticosteroids frequently have a nonspecific effect, decreasing pro-inflammatory and anti-inflammatory responses, which might be detrimental in conditions requiring specialized modulation of immune pathways [75,77].

### 5.3. Cytokine-Specific Inhibitors and Monoclonal Antibodies

Targeted therapy using monoclonal antibodies (mAbs) or small-molecule inhibitors against specific cytokines, such as TNF-α, IL-1β, and IL-6, offers a more precise way of controlling neuroinflammation. Anti-TNF-α treatments, such as infliximab and adalimumab, have shown benefits in systemic inflammatory illnesses such as rheumatoid arthritis and Crohn’s disease. Their potential for application to neuroinflammatory conditions is being investigated [13,78,79]. Anakinra, an IL-1β antagonist, has shown neuroprotective effects in preclinical stroke and traumatic brain damage studies. However, outcomes in regard to chronic neurodegenerative illnesses are inconsistent [80,81]. Despite these advancements, many cytokine inhibitors have difficulty crossing the BBB, and their systemic immunosuppressive effects raise questions regarding infection risks and long-term safety [57].

### 5.4. Small-Molecule Inhibitors

Small-molecule inhibitors that target intracellular signaling pathways involved in cytokine generation, such as the JAK/STAT pathways, serve as another option for controlling neuroinflammation. JAK inhibitors, such as tofacitinib and ruxolitinib, have been used successfully in treating autoimmune illnesses and are being studied for their capacity to reduce neuroinflammation in disorders such as MS and AD [18,82,83]. These inhibitors can be delivered orally and penetrate the BBB more easily than bigger biologic compounds. However, like with other immunosuppressive medications, they increase the risk of opportunistic infections and may have off-target effects on other immune-regulatory mechanisms, necessitating careful dose management and monitoring in clinical settings [84,85,86].

### 5.5. Emerging Therapies: Nanotechnology and Gene-Based Approaches

Recent advancements in the delivery of drugs and molecular biology have created new opportunities for targeting neuroinflammation with high accuracy. Nanotechnology-based delivery systems, such as liposomes, polymeric nanoparticles, and lipid nanoparticles, can be designed to cross the BBB, allowing anti-inflammatory medicines or cytokine inhibitors to be delivered directly to inflamed brain regions. These techniques increase drug stability, reduce off-target effects, and improve therapeutic efficacy [87,88].

Gene therapy and RNA interference (RNAi) are also emerging as promising approaches to controlling cytokine activity in the CNS. In these techniques, viral vectors or RNA molecules are used to transfer genes that boost anti-inflammatory signaling or inhibit specific pro-inflammatory cytokines, enabling long-term control of the inflammatory response [89,90,91]. Preclinical findings suggest that utilizing RNAi to mute TNF-α and IL-1β production can reduce neuroinflammation and preserve neuronal integrity in neurodegenerative models [92,93]. While these therapies are still experimental, they provide a promising horizon for the precise and long-term treatment of neuroinflammatory diseases.

### 5.6. Personalized Medicine and Biomarker-Guided Therapy

Personalized medicine, in which therapy is tailored based on an individual’s distinct inflammatory profile, is a growing trend in neuroinflammatory treatment. Biomarkers such as C-reactive protein (CRP), cytokine levels, and inflammation-related neuroimaging measurements are employed to identify individuals who could benefit from focused anti-inflammatory therapy. Personalized techniques can enhance therapeutic results by targeting interventions to patients most likely to benefit from cytokine regulation [94,95,96].

Current techniques for controlling inflammation in brain illnesses show a trend toward more precise, focused therapies that reduce systemic adverse effects while effectively modifying neuroinflammatory pathways. From classic anti-inflammatory drugs to new cytokine inhibitors and cutting-edge delivery technologies, these treatments are at the forefront of combating the immune-driven disorders that underpin many CNS diseases. As new technology and biomarker-guided medicines advance, more effective and individualized treatment regimens that address both the complexity and chronicity of neuroinflammation in brain illnesses will become possible.

## 6. Emerging Treatment Strategies for Cytokine-Mediated Inflammation

Recent advancements in neuroscience and immunology have opened the door for new therapeutic approaches to cytokine-mediated inflammation in brain diseases. These developing techniques aim to tune the immune response more precisely, reduce systemic adverse effects, and provide long-term therapeutic advantages. These approaches, from biologics to advanced gene-editing methods, reflect an increasing awareness of the complex link between the immune system and neurological health.

### 6.1. Biologics Targeting Cytokine Pathways

Biologics, such as mAbs and receptor antagonists, offer a transformational approach to targeting specific cytokines involved in neuroinflammation. Adalimumab and etanercept, TNF-α inhibitors, are being repurposed for neurological uses. Preclinical and clinical investigations show promise in decreasing inflammation-associated neuronal damage [97,98,99]. Similarly, IL-6 inhibitors (e.g., tocilizumab) and IL-1 receptor antagonists (e.g., anakinra) are being studied in relation to diseases such as MS, AD, and stroke [100,101]. These medicines provide high accuracy in modulating specific inflammatory pathways but require optimization to overcome obstacles such as BBB permeability and off-target effects.

### 6.2. RNA-Based Therapies

RNA-based therapeutics, such as RNAi and antisense oligonucleotides (ASOs), provide highly targeted strategies for lowering cytokine expression. RNA-based techniques can reduce neuroinflammation and protect neurons by silencing genes that produce pro-inflammatory cytokines, including IL-1β and TNF-α. For example, ASO treatments targeting SOD1 effectively lowered neuroinflammation and slowed disease progression in amyotrophic lateral sclerosis (ALS) [102,103,104]. These medications are still experimental, but they have tremendous potential for treating inflammation-driven brain diseases.

### 6.3. Gene Editing and CRISPR Technology

Gene-editing tools, particularly CRISPR-Cas9, are being investigated for their potential to change genes implicated in cytokine production and inflammatory pathways. CRISPR can decrease pro-inflammatory signaling for an extended period by targeting cytokine genes or their upstream regulators. Early preclinical research indicated the feasibility of utilizing CRISPR to reduce neuroinflammation in models of AD and TBI [105,106,107]. However, issues such as off-target effects and the development of safe delivery techniques must be solved before these ideas can be put into clinical practice.

### 6.4. Modulation of the Gut–Brain Axis

The gut–brain axis, a bidirectional communication network that includes the immunological, neurological, and endocrine systems, is crucial in regulating neuroinflammation [108]. Dysbiosis of the gut microbiota has been associated with increased cytokine production and the worsening of brain illnesses such as PD and depression [109,110]. Emerging therapeutics, such as probiotics, prebiotics, and fecal microbiota transplantation, are designed to restore gut balance. These techniques have shown potential in modifying systemic and central inflammation by lowering circulating pro-inflammatory cytokine levels and increasing anti-inflammatory responses.

### 6.5. Neuroprotective Peptides and Small Molecules

Small compounds and neuroprotective peptides are being produced to combat the adverse effects of cytokines in the brain. For example, cytokine receptor peptides can operate as decoys, binding pro-inflammatory cytokines and blocking them from interacting with cellular targets [111,112]. Furthermore, small-molecule inhibitors of JAK pathways, such as baricitinib, are being explored for their capacity to block intracellular cytokine signaling, bringing fresh hope to patients with autoimmune and neurodegenerative illnesses [18,83].

### 6.6. Cellular Therapies

Cell-based therapies, particularly those involving the utilization of mesenchymal stem cells (MSCs), have received attention due to their immunomodulatory capabilities. MSCs produce anti-inflammatory cytokines, promote tissue healing, and inhibit the generation of pro-inflammatory mediators in the CNS. Clinical investigations have shown that MSCs can reduce neuroinflammation and improve functional results for disorders such as MS and stroke [113,114]. Advancements in stem cell engineering may improve their therapeutic efficacy and specificity.

### 6.7. Personalized and Biomarker-Guided Therapies

The increasing availability of biomarkers that represent cytokine activity in the CNS enables the development of tailored treatment regimens. Biomarker-guided techniques can allow clinicians to adapt medicines based on an individual’s inflammatory profile, increasing efficacy while reducing side effects. Cytokine profiling in cerebrospinal fluid (CSF) and advanced neuroimaging modalities can help identify patients who will benefit most from specific anti-inflammatory therapies [115,116]. These tailored techniques are beneficial in the treatment of several disorders, such as depression and schizophrenia, where inflammation varies significantly between individuals.

Emerging therapeutics, such as RNAi and CRISPR-based gene editing, are being investigated to silence cytokine-related genes, providing a highly specific approach to modulating the immune response in neuroinflammation. Addressing comorbidities is a critical component of neuroinflammation management. Effective management of hypertension and diabetes can lower levels of systemic inflammatory markers such as TNF-α and IL-6, reducing their effects on the CNS. Integrative therapy techniques that address both primary CNS inflammation and associated diseases may lead to better outcomes in neuroinflammatory disorders. As research advances, integrating these methods into clinical practice offers enormous potential for improving the lives of individuals suffering from inflammation-driven brain illnesses.

## 7. Novel Approaches to Drug Delivery in the Brain

The efficient treatment of brain illnesses frequently confronts a fundamental challenge: overcoming the BBB. This carefully regulated barrier shields the brain from dangerous compounds while restricting the entry of therapeutic medicines, such as those targeting cytokine-mediated inflammation. Traditional drug delivery strategies are typically ineffective due to inadequate BBB penetration and systemic adverse effects. In response, novel ways of improving the delivery of drugs to the brain have emerged, allowing for more precise and effective therapies.

### 7.1. Nanotechnology-Based Drug Delivery

Nanotechnology has transformed CNS drug delivery by allowing therapeutic substances to traverse the BBB in a regulated and targeted manner. Nanoparticles, liposomes, dendrimers, and micelles are among the most extensively researched systems for brain-targeted drug delivery. These nanoscale carriers can encapsulate cytokine inhibitors, biologics, or small compounds, preserving them from degradation while increasing their solubility and bioavailability [117]. Lipid nanoparticles have successfully been used to deliver anti-inflammatory medicines, including siRNA targeting TNF-α, in animal models of neurodegenerative disorders [118].

Nanoparticles have shown potential in the delivery of cytokine inhibitors. Recent breakthroughs in surface-modified nanoparticles have enabled them to cross the BBB via receptor-mediated transcytosis, allowing for targeted medication delivery in diseases such as AD and MS [119]. Functionalizing nanoparticles with ligands such as transferrin, apolipoproteins, or antibodies enables receptor-mediated transport across the BBB, enhancing selectivity and minimizing off-target effects. Furthermore, the optimal nanocarrier contains two ligands: one aids in passage through the BBB, and the other targets a specific region of the brain by enabling rapid drug release upon arrival, triggered by pH changes or enzymatic activity, thereby preventing premature release [120]. As a matter of fact, nanoparticles can be tailored for sustained release, which ensures a prolonged therapeutic impact and reduces the frequency of delivery.

### 7.2. Ultrasound-Enhanced Delivery

Magnetic-resonance-image-guided focused ultrasound (FUS) in combination with microbubbles has emerged as a powerful approach for temporarily and noninvasively opening the BBB, allowing for localized medication delivery to the brain. In this approach, FUS-transmitted pressure waves oscillate systematically administered gas-encasing microtubules and coincide at a precise focal spot with millimeter-level accuracy. The endothelial cells’ tight junctions are broken by this mechanical action, which permits therapeutics to enter the FUS-targeted focal region methodically [121,122]. This technique has been used in preclinical and early clinical research to deliver anti-inflammatory medicines and monoclonal antibodies targeting cytokines such as IL-1β and TNF-α in neurodegenerative illnesses such as AD [121].

FUS provides the advantage of precise geographic targeting, reducing systemic side effects and off-target repercussions. Ongoing clinical trials are studying its potential to improve the delivery of biologics, RNA-based medicines, and nanocarriers to specific brain regions.

### 7.3. Intranasal Drug Delivery

Intranasal delivery bypasses the BBB by delivering a drug directly to the brain via the olfactory and trigeminal neurons. Although the nose-to-brain pathway is currently poorly understood, numerous recent studies have proposed some important potential pathways. Physiologically, olfactory neurons are directly involved in olfactory transmission through nasal cavities leading to the brain. This pathway enables the direct transmission of a drug to the brain without requiring it to go through systematic circulation [123]. Other potential strategies for drug transport through olfactory epithelial cells include endocytosis and simple diffusion, such as transcellular and paracellular approaches via cell junctions [123]. Intranasal drug delivery has received attention due to its ability to directly provide cytokine inhibitors and other therapeutic medicines to inflammatory locations in the CNS [124]. For example, intranasal administration of IL-1 receptor antagonists has been effective in preclinical models of traumatic brain damage and stroke [125,126].

The aging of the population is closely associated with an increase in the prevalence of CNS disorders, including neuropsychiatric, neoplastic, and neurodegenerative diseases. Since the BBB prevents 98% of small molecules and 100% of macromolecule-type therapeutic agents from penetrating brain tissue, intranasal delivery may be a great approach in this respect [127]. Intranasal vaccinations have gained attention as a result of the deaths caused by the coronavirus disease (COVID-19) worldwide. A thorough analysis that covers all trends in intranasal studies is lacking, despite the fact that a number of excellent reviews, including those on intranasal oxytocin research, intranasal COVID-19 vaccines, and intranasally functionalized polymeric nanomaterials, have concentrated on particular facets of intranasal delivery [127]. The main obstacle currently preventing the advancement of intranasal drug delivery is the absence of an effective and efficient delivery system that can more closely mimic the human device for olfactory mucosa targeting in animal studies [128]. It is very crucial to validate in vitro study findings through an in vivo context.

Several intranasal–brain drug delivery studies using rodents are actually at the preclinical level according to previous reports; however, the noninvasive intranasal technique has the potential to allow quick drug administration, making it ideal for acute diseases [123,129,130,131]. Formulation advancements, such as using mucoadhesive nanoparticles or gels, have increased medication retention in the nasal cavity and brain-targeted administration.

### 7.4. Exosome-Based Delivery

Exosomes, naturally occurring nanovesicles released by cells, have emerged as attractive drug delivery carriers due to their biocompatibility, capacity to traverse the BBB, and intrinsic cell-targeting capabilities. Exosomes can be designed to deliver cytokine inhibitors, RNA-based therapies, or neuroprotective medicines directly to inflamed brain areas [132,133,134]. Exosomes have low cytotoxicity and immunological responsiveness because they come from a person’s own cells. Exosomes move through extracellular matrices after departing their origin cell in order to release their cargo and initiate cell-to-cell communication inside the recipient cell [135]. Preclinical studies have demonstrated that the exosome-mediated delivery of anti-inflammatory medicines can reduce neuroinflammation and enhance outcomes in animal models of MS and PD.

One significant advantage of exosomes is their capacity to avoid immune recognition, lowering the chance of unwanted reactions. Furthermore, exosome engineering enables the targeting of specific cell types, such as microglia or astrocytes, which play critical roles in cytokine-mediated inflammation.

### 7.5. Polymer-Based Drug Delivery Systems

Biodegradable polymers, such as poly(lactic-co-glycolic acid) (PLGA), are used to create implanted or injectable devices for continuous drug administration in the brain. These polymers can encapsulate anti-inflammatory drugs and release them over time, ensuring consistent therapeutic doses are provided and reducing systemic exposure. Implantable polymer wafers filled with cytokine inhibitors were investigated for treating glioblastoma-associated inflammation [136].

In addition to implants, injectable hydrogels of biocompatible polymers can be administered directly to inflamed brain areas, providing targeted treatment with low systemic damage. These methods are promising for chronic neuroinflammatory illnesses that necessitate long-term cytokine regulation.

### 7.6. Gene-Delivery Systems

Gene therapy techniques involving viral vectors, such as adeno-associated viruses (AAVs) or lentiviruses, are being developed to transfer genes that encode anti-inflammatory cytokines or cytokine inhibitors. These vectors can have long-term therapeutic effects by increasing the production of therapeutic proteins in the CNS [137,138]. For example, AAVs that carry IL-10, an anti-inflammatory cytokine, have shown promise in lowering neuroinflammation in PD and MS animal models.

Nonviral gene delivery technologies, such as lipid nanoparticles and electroporation techniques, are also being investigated to address safety concerns about viral vectors. These methods allow for temporary gene expression, which provides flexibility in modulating therapeutic effects.

### 7.7. Targetting Upstream Inflammasome Activation

NOD-like receptors (NLR), also referred to as nucleotide-binding leucine-rich repeat-containing receptors, are cytosolic sensors that react to pathogen-associated molecular patterns (PAMPs), which are connected to different microorganisms and danger-associated molecular patterns (DAMPs), which are generated during tissue-based injuries [139]. Upon sensing a DAMP or PAMP, a multimeric protein complex, which is known as an inflammasome, is formed by association with NLR, an adaptor protein ASC (apoptosis-associated speck-like protein containing a C terminal caspase recruitment domain [CARD]), and pro caspase-1 [139]. This macromolecular complex facilitates proximity-induced autoactivation of caspase-1 to promote the maturation of pro-inflammatory cytokines IL-1β and IL-18 [140]. Numerous neurodegenerative diseases, including MS, ALS, PD, and AD, have been linked to inflammasome activation [141].

As a new pharmacological approach to specifically altering inflammasome activation in pathological circumstances, upstream targeting of inflammasome pathways has recently attracted attention [140]. AIM2 (absent in melanoma 2), NLRP1 (NLR family, pyrin domain containing 1), and NLRP3 (NLR family, pyrin domain-containing 3), NLRC4 (NLR family, CARD domain-containing protein 4) are examples of inflammasome-forming proteins that are potential ideal therapeutic targets because they are essential in mediating the release of the cytokines IL-1β and IL-18 [139,140].

Although NLRP1/3 is the most prevalent inflammasome in the CNS, NLRP3 is the one that has been studied the most, and it has been implicated in neurodegenerative diseases [141]. Dipeptidyl peptidase 9 and thioredoxin are examples of endogenous inhibitors that have been found to interact with NLRP1, raising the prospect of creating small-molecule medications that target NLRP1 to treat neurodegenerative diseases. Recent reports indicate that some biological and small-molecule inhibitors that target the NLRP1 inflammasome to treat neurodegenerative diseases are progressing to preclinical testing [142]. The most powerful and NLRP3-specific of the direct NLRP3 inhibitors is the diaryl sulfonylurea compound MCC950, which showed therapeutic efficacy in several preclinical immunopathological models, such as experimental autoimmune encephalomyelitis (EAE), which is a disease model of MS, and rat models of AD and PD [143,144,145].

Prolonged and excessive inflammasome activation can cause inflammation. Also, targeting IL-1β and IL-18, two byproducts of the inflammasome, is a limited strategy that can result in widespread immune suppression [146]. In contrast to the general inhibition of inflammasome products, future research is needed to develop therapeutic approaches that target tissue-specific inflammasome subtypes, and new therapies that target the sensor, adaptor, and effector components of inflammasomes can offer the clinical possibility of regulating inflammasome activation at several stages, improving the outcomes of neurological disorders linked to inflammasomes [147].

### 7.8. Combination Strategies

Emerging evidence suggests that combining delivery strategies can improve therapeutic efficacy. To improve BBB penetration and target specificity, nanoparticles containing anti-inflammatory drugs can be delivered intranasally or via FUS. Similarly, exosomes functionalized with ligands for receptor-mediated transport can increase the delivery of RNA-based medicines to specific brain areas.

Drug delivery technology advancements alter the therapy landscape for brain illnesses caused by cytokine-mediated inflammation. By circumventing the BBB’s obstacles, these innovative techniques enable precise, targeted, and effective therapeutic administration while decreasing systemic toxicity and improving clinical outcomes. As these tactics progress, they can transform the management of neuroinflammatory illnesses and enhance patients’ lives.

## 8. Potential Biomarkers for Monitoring Inflammatory Response

The discovery of reliable biomarkers for monitoring inflammatory responses in brain illnesses is essential for improving diagnosis, therapy efficacy, and disease management. Biomarkers that reflect the dynamics of cytokine-mediated inflammation in the CNS can inform individualized therapy tactics, allowing for early intervention and real-time assessment of treatment effects. Advances in proteomics, transcriptomics, and neuroimaging have increased the repertory of possible biomarkers for inflammatory brain diseases.

### 8.1. Cytokines and Chemokines in Cerebrospinal Fluid (CSF) and Plasma

Cytokines and chemokines are critical mediators of neuroinflammation and direct indications of immunological activity in the CNS. Pro-inflammatory cytokine levels, including TNF-α, IL-6, and IL-1β, are continuously elevated in patients with AD, MS, and depression [12]. These cytokines can be tested in CSF or blood, but CSF frequently provides a more accurate depiction of CNS-specific inflammation.

Anti-inflammatory cytokines, such as IL-10, can also be used as biomarkers to indicate the activation of compensatory mechanisms to reduce inflammation [148]. The pro- and anti-inflammatory cytokine balance can provide insight into disease development and therapy responses.

### 8.2. Microglial and Astrocytic Activation Markers

Microglia and astrocytes are significant causes of neuroinflammation, and their activation can be detected via soluble biomarkers or imaging techniques. Soluble triggering receptor expressed on myeloid cells 2 (sTREM2), a microglial activation marker, can be detected in CSF and corresponds with neuroinflammatory activity in disorders including AD and PD [149,150]. Similarly, glial fibrillary acidic protein (GFAP), a marker of astrocytic activation, has been found to be a possible biomarker for neuroinflammatory illnesses such as TBIs and MS [151,152].

### 8.3. Neurofilament Light Chain (NfL)

Neurofilament light chain (NfL) is a structural protein released into the cerebrospinal fluid and bloodstream following neuronal injury; this process is typically induced by inflammation. Elevated NfL levels have been linked to axonal damage in neuroinflammatory diseases, including MS, ALS, and HIV-associated neurocognitive deficits [153,154]. NfL is a promising non-invasive biomarker for monitoring disease progression and medication efficacy, enabled by ultrasensitive detection techniques such as single-molecule array (SIMOA) tests.

### 8.4. Immune-Cell-Derived Extracellular Vesicles

Immune cells, such as microglia and macrophages, secrete extracellular vesicles (EVs) containing cytokines, chemokines, and other inflammatory molecules. These vesicles can pass through the BBB and be identified in peripheral blood, offering a non-invasive way to monitor CNS inflammation [155,156]. The molecular cargo of EVs, which contains particular miRNAs, can reflect the brain’s inflammatory state and provide valuable insights into the mechanisms behind neuroinflammatory diseases.

### 8.5. Metabolites and Lipid Mediators

Metabolites and lipid mediators involved in the inflammatory cascade are being identified as biomarkers of neuroinflammation. For example, kynurenine, a tryptophan metabolite involved in the immune response, has been linked to depression and schizophrenia, demonstrating that the kynurenine pathway is activated in response to inflammation [157,158]. Similarly, lipid mediators such as prostaglandins and resolvins exhibit pro- and anti-inflammatory activity and can be tested in CSF and plasma.

### 8.6. Neuroimaging Biomarkers

Imaging breakthroughs such as positron emission tomography (PET) and magnetic resonance imaging (MRI) have identified neuroinflammation biomarkers in vivo. PET tracers targeting translocator protein 18 kDa (TSPO), a marker of activated microglia, allow for spatial and temporal resolution of inflammatory processes in the brain [159,160,161,162]. Furthermore, MRI-based assessments of brain volume, white matter abnormalities, and iron deposition are used as indirect indicators of persistent neuroinflammation in illnesses such as MS and AD.

### 8.7. Genomic and Transcriptomic Markers

Genomic and transcriptome profiling have revealed gene expression patterns linked to inflammatory responses in brain diseases. Transcriptomic analysis of peripheral blood mononuclear cells (PBMCs) has revealed inflammatory gene signatures, including those encoding cytokines, chemokines, and their receptors, that are associated with disease activity in disorders such as schizophrenia and bipolar disorder [163,164]. These fingerprints have the potential to identify patient subgroups and predict therapy responses.

### 8.8. The Gut–Brain Axis Biomarkers

The gut–brain axis is becoming more widely recognized for regulating CNS inflammation. Dysbiosis of the gut microbiota has been linked to increased systemic and CNS pro-inflammatory cytokine levels [108]. Biomarkers such as lipopolysaccharides (LPS) and short-chain fatty acids (SCFAs) can shed light on how the gut contributes to neuroinflammatory illnesses such as PD and depression [109,165].

Identifying and validating biomarkers for cytokine-mediated inflammation in brain illnesses has significant potential for improving precision medicine. These biomarkers are substantial tools for coping with the issues posed by neuroinflammatory illnesses because they provide insights into disease mechanisms, guide therapeutic options, and enable therapy efficacy monitoring. Future research should focus on integrating several biomarker modalities to capture the complexities of inflammatory processes and improve therapeutic outcomes.

## 9. Preclinical and Clinical Trials

The development of therapeutics targeting cytokine-mediated inflammation in brain diseases has made significant progress thanks to rigorous preclinical and clinical trials. These studies evaluate the safety, efficacy, and mechanisms of developing medicines, giving crucial information about their therapeutic potential. This section summarizes significant discoveries from preclinical investigations and clinical trials investigating cytokine regulation in neuroinflammatory disorders.

### 9.1. Preclinical Studies

Preclinical research is the foundation of therapeutic discovery, providing essential insights into disease mechanisms and the efficacy of new therapies when applied to animal models.

Cytokine Inhibitors: Animal models of AD, PD, and MS have shown that cytokine inhibitors effectively reduce neuroinflammation. In transgenic mouse models of AD, monoclonal antibodies targeting TNF-α improved cognitive deterioration [31]. IL-1β inhibitors improved motor function and reduced glial activation in animal models of PD [166]. Recent Phase II studies with IL-1 receptor antagonists showed considerable cognitive improvement in patients with moderate AD, implying that regulating cytokine signaling can slow disease development [167].

Gene Therapy: Preclinical experiments in which adeno-associated viral (AAV) vectors were used to transmit anti-inflammatory cytokines such as IL-10 have shown promise in regard to chronic neuroinflammatory disorders, including ALS and Huntington’s disease, by lowering microglial activation and neuronal damage [168].

Nanoparticle-Based Delivery: In preclinical studies, nanoparticle-based delivery methods improved the CNS bioavailability of cytokine inhibitors. For example, lipid nanoparticles containing siRNA targeting IL-6 dramatically reduced inflammatory indicators and neuronal death in stroke models [169].

### 9.2. Clinical Trials

Clinical trials are critical for turning preclinical results into safe and effective treatments for patients. Several clinical trials have examined cytokine-modulating therapy in a variety of brain diseases.

TNF-α Inhibitors: TNF-α inhibitors such as infliximab and etanercept, once used for autoimmune illnesses like rheumatoid arthritis, are now utilized to treat CNS disorders. A pilot study of etanercept in post-stroke patients found that it improved motor function and mood by reducing neuroinflammation [97]. However, larger randomized controlled trials are necessary to corroborate these findings.

IL-1β Antagonists: Anakinra, an IL-1 receptor antagonist, was tested in TBI patients and found to reduce systemic inflammatory markers while improving clinical outcomes in early-phase trials [81]. Ongoing trials are evaluating its potential in neurodegenerative illnesses such as AD.

IL-6 Blockade: Tocilizumab, an IL-6 receptor antagonist, has been studied in relation to depression and schizophrenia. A randomized trial on treatment-resistant depression reported improvements in depressive symptoms, corresponding with lower levels of peripheral inflammatory markers [170,171].

Combination Therapies: Clinical investigations have indicated that combining anti-inflammatory medicines with neuroprotective techniques can be effective. A phase II trial with minocycline and an IL-1β inhibitor in MS patients found a synergistic benefit, lowering lesion development and neuroinflammatory indicators [172].

### 9.3. Immune Modulation in Specific Disorders

Alzheimer’s Disease: Several trials have investigated immune-modulating drugs in relation to AD. Solanezumab, an anti-amyloid monoclonal antibody that does not directly target cytokines, has shown promise in decreasing levels of neuroinflammatory indicators in CSF [173,174]. Trials that combine amyloid-targeting treatments with anti-inflammatory medications are ongoing.

Multiple Sclerosis: Phase III trials have proven that therapies that modify cytokine signaling pathways, such as fingolimod and siponimod, significantly reduce relapse rates and lesion volume in MS patients [175].

Traumatic Brain Injury: Clinical investigations of stem cell therapy have revealed that mesenchymal stem cells can reduce cytokine-mediated inflammation and increase recovery among TBI patients. These benefits are believed to be mediated by the release of anti-inflammatory cytokines such as IL-10 [176].

Table 2 summarizes clinical trials targeting cytokine pathways. Despite promising outcomes, many clinical trials encounter obstacles such as patient heterogeneity, biomarker variability, and the ability of a drug to penetrate through the BBB. To overcome these problems, ongoing trials are looking into stratified patient populations based on inflammatory biomarkers and improved drug delivery technologies such as nanoparticles and targeted ultrasound [175,177].

Emerging medicines that target larger cytokine networks and are implemented using precision medicine approaches show promise for improving clinical outcomes. Furthermore, adaptive trial designs and the utilization of real-world data are expected to hasten the development of successful cytokine-targeting medicines. Table 3 summarizes current and emerging therapies.

The translational research pipeline for cytokine-modulating therapeutics has advanced significantly, with multiple preclinical and clinical trials showing efficacy in lowering neuroinflammation and improving clinical outcomes. Continued efforts to enhance drug delivery, validate biomarkers, and integrate novel therapeutic techniques should pave the path for successful treatments for brain illnesses defined by cytokine-mediated inflammation.

## 10. Future Directions and Perspectives

The changing landscape of cytokine-mediated inflammation research regarding brain diseases provides an opportunity to develop novel treatments and enhance patient outcomes. Despite tremendous progress, hurdles remain, such as understanding the complexities of cytokine networks, overcoming medication delivery barriers, and identifying specific biomarkers to guide therapy. This section discusses significant topics of future research and developing viewpoints in the discipline.

### 10.1. Expanding the Understanding of Cytokine Pathways

Understanding cytokine signaling pathways and their functions in neuroinflammatory disorders is critical. Future studies should focus on understanding the complex relationships between cytokines, glial cells, and neurons. Advanced single-cell and spatial transcriptomics can provide insights into cell-specific cytokine responses, allowing the identification of new therapeutic targets [4,178,179]. Future studies must also focus on the interactions between systemic comorbidities and neuroinflammation. Understanding how diseases such as hypertension and diabetes affect cytokine pathways could help guide the development of integrated treatment strategies targeting systemic and CNS-specific inflammation, resulting in more comprehensive management of brain illnesses.

### 10.2. Precision Medicine Approaches

Treatments tailored to individual patients based on their inflammatory profiles show significant promise. Advances in omics technologies, such as proteomics, genomes, and metabolomics, can help stratify individuals into subgroups with different cytokine dysregulation patterns. Machine learning algorithms used for multi-omics data may allow the prediction of therapeutic responses, paving the way for precision medicine in neuroinflammatory illnesses [180,181,182]. Advances in computational modeling and AI-driven predictive analytics are enabling tailored cytokine profiling for CNS illnesses, which could guide the development of patient-specific therapy methods [183].

### 10.3. Novel Therapeutic Targets and Agents

Identifying novel therapeutic targets beyond TNF-α and IL-6 is crucial for further research. Emerging targets include anti-inflammatory pathways, particularly those involving the cytokines IL-37 and IL-38, which have exhibited protective effects in preclinical models of neuroinflammation [13,184,185,186]. Small compounds targeting upstream regulators, such as NF-κB and JAK/STAT pathways, are being developed to modify cytokine signaling further.

### 10.4. Innovative Drug Delivery Systems

Overcoming the challenge of delivering cytokine-modulating medicines across the BBB remains a significant goal. Focused ultrasound, lipid nanoparticles, and receptor-mediated transport systems are technologies that have the potential to improve CNS medication delivery [117,169,187]. Combining these technologies with real-time imaging may increase the precision targeting of inflamed brain areas. Table 4 summarizes novel drug delivery technologies.

### 10.5. Biomarkers for Real-Time Monitoring

Creating dynamic and reliable biomarkers is essential for determining disease progression and therapeutic efficacy. Future research should verify the effectiveness of non-invasive biomarkers, such as blood-based cytokine profiles and extracellular vesicle-derived inflammatory mediators, as real-time monitoring tools [188]. Furthermore, combining biomarker data with neuroimaging results may allow more thorough comprehension of neuroinflammatory processes. Table 5 summarizes biomarkers in neuroinflammation.

### 10.6. Combination Therapies

Given the diverse nature of neuroinflammation, combination treatments that target many cytokines or pathways at once may be more effective. Anti-inflammatory medications, for example, might be used in conjunction with neuroprotective therapies, such as antioxidants or synaptic modulators, to synergistically target both inflammation and neuronal damage [111,189]. Future clinical trials should investigate and evaluate these combinations’ long-term safety and efficacy.

### 10.7. Role of the Gut-Brain Axis

The gut–brain axis is increasingly recognized as a key regulator of neuroinflammation. The body of research into microbiome-targeted therapeutics, such as probiotics, prebiotics, and fecal microbiota transplantation, is quickly expanding. These treatments are intended to balance the gut microbiota and reduce systemic inflammation, factors that contribute to CNS diseases [190]. Researching the impact of gut-derived cytokines on brain health is a critical future direction.

### 10.8. Neuroimmune Crosstalk and Aging

Aging is a significant risk factor for several neuroinflammatory illnesses, including AD and PD. Understanding how age affects neuroimmune interaction and predisposes the brain to chronic inflammation is critical. Research on immunosenescence and age-associated alterations in cytokine signaling may guide strategies for decreasing age-related neuroinflammation [191,192].

### 10.9. Regulatory and Ethical Considerations

As sophisticated medicines and techniques such as gene editing and cytokine-based biologics begin to undergo clinical trials, ethical and regulatory issues must be addressed. Critical challenges include ensuring equal access, monitoring long-term hazards, and developing robust frameworks for gene therapy in brain illnesses. Collaboration among scientists, doctors, regulators, and patient advocates is crucial for properly developing and implementing these therapies [193,194,195].

The future of cytokine-mediated inflammation in brain illnesses depends on interdisciplinary collaboration and the integration of cutting-edge technologies. By tackling drug delivery, biomarker discovery, and patient heterogeneity, researchers might pave the way for more effective and tailored treatments. Continuous investment in preclinical and clinical research, combined with improvements in precision medicine and medication delivery, has enormous potential to change the management of neuroinflammatory diseases.

## 11. Conclusions

Cytokine-mediated inflammation is critical in the pathophysiology of many brain disorders, including neurodegenerative diseases, psychiatric problems, and acute traumas. Advances in understanding cytokines’ complicated interplay inside the CNS have shown their dual role as mediators of neuroprotection and neurodegeneration. This finding emphasizes the ability of cytokine-targeting therapy to control inflammation and restore neuronal function.

Preclinical and early clinical trials have shown promise for emerging therapeutic techniques such as using cytokine inhibitors, immune-modulating biologics, and small compounds targeting inflammatory signaling pathways. Drug delivery innovations, such as nanoparticle-based systems and targeted ultrasound, have made it more feasible to transport therapeutic drugs across the BBB. Meanwhile, identifying and validating biomarkers, such as cytokine profiles and imaging modalities, advance the capacity to monitor disease progression and therapy responses in real-time.

Despite these advances, difficulties remain. Patient population heterogeneity, difficulty in transferring preclinical findings to clinical settings, and the complexities of CNS-specific inflammation all call for a more personalized approach. Precision medicine, guided by multi-omics and machine learning methods, provides a road to tailored therapeutic approaches. Furthermore, combining neuroinflammation research with insights into the gut–brain axis, aging, and neuroimmune interaction could lead to new therapeutic strategies.

Collaboration across disciplines will become increasingly important as this area advances. Collaborations between researchers, physicians, and industry stakeholders can speed up the translation of findings into successful medicines. Ethical considerations and fair access to modern therapies must also inform the development and implementation of novel interventions.

In conclusion, while hurdles remain, the expanding corpus of data on cytokine-mediated inflammation in brain diseases signals a new era in treating these debilitating conditions. The potential to revolutionize patient outcomes and improve quality of life can be within our grasp if we harness emerging technology and encourage interdisciplinary collaboration.

## Figures and Tables

**Table 1:** Overview of cytokines’ roles in brain disorders.

Disorder	Key Cytokines	Pathophysiological Roles	Potential Therapeutic Targets
AD	IL-1β, TNF-α, IL-6	Promote amyloid aggregation, neurotoxicity	Anti-TNF therapies (e.g., infliximab)
MS	IFN-γ, IL-17, TNF-α, IL-6	Activate immune cells, demyelination	Anti-IL-17 monoclonal antibodies
MDD	IL-6, TNF-α, IFN-γ, IL-17, IL-10, IL-1β	Induce hypothalamic-pituitary-adrenal axis dysregulation, neuronal apoptosis	Anti-IL-6 agents (e.g., tocilizumab)
PD	IL-1β, TNF-α, IL-6, IFN-γ	Microglial activation, dopaminergic neuron loss	Microglia inhibitors
TBI	IL-1β, IL-10, TNF-α, IL-6	Acute inflammation, secondary injury cascade	Cytokine modulators

**Table 2:** Clinical trials targeting cytokine pathways.

Therapy	Condition	Phase	Key Findings
Tocilizumab	Depression	Phase II	Reduced inflammatory markers, improved mood
Infliximab	AD	Phase II	Attenuated neuroinflammation, early efficacy
JAK Inhibitors (Tofacitinib)	MS	Phase I	Reduced immune cell infiltration
IL-17 Monoclonal Antibody	MS	Phase III	Decreased relapse rates
Microbiome Therapies	PD	Phase I	Modulation of systemic inflammation

**Table 3:** Summary of current and emerging therapies.

Therapy Type	Example Agents	Targeted Cytokines	Status (Preclinical/Clinical)
Biologics	Infliximab, Tocilizumab	TNF-α, IL-6	Clinical Phase II–III
Small Molecules	JAK inhibitors (ruxolitinib)	JAK/STAT pathway	Clinical Phase I–II
Antisense Oligonucleotides	N/A	IL-1β	Preclinical
Gene Therapy	CRISPR/Cas9	TNF-α and IL-6	Preclinical
Microbiome Therapies	Probiotics, Prebiotics	Gut-derived cytokines	Clinical Phase I

N/A: not applicable.

**Table 4:** Novel drug delivery technologies.

Technology	Mechanism	Advantages	Challenges
Nanoparticles	Targeted drug release	High specificity, BBB penetration	Variability in BBB uptake
Focused Ultrasound	Temporary BBB disruption	Non-invasive, real-time control	Risk of tissue damage
Liposomes	Encapsulation of drugs	Reduced systemic toxicity	Limited CNS targeting
Receptor-Mediated Transport	Ligand–receptor interaction	Enhanced BBB transport	Requires specific ligand design
Hydrogels	Localized release	Sustained delivery at target site	Limited mobility for CNS-wide effects

**Table 5:** Biomarkers in neuroinflammation.

Biomarker Type	Source (CSF/Blood)	Diagnostic Use	Current Status
Cytokines (IL-6, IL-1β)	Blood	Monitor systemic inflammation	Validated for clinical use
Extracellular Vesicles	CSF/Blood	Indicator of CNS injury	Experimental
Neurofilament Light (NfL)	CSF/Blood	Axonal damage detection	Approved for Alzheimer’s disease monitoring
Proteomic Signatures	Blood/CSF	Disease-specific inflammatory profile	Under investigation
Imaging Biomarkers	PET scans, MRI	Visualization of neuroinflammation	Validated for research

## Data Availability

Data sharing is not applicable.

## Abbreviations

BBB: blood–brain barrier; CNS: central nervous system; NF-κB: nuclear factor-kappa B; MAPK: mitogen-activated protein kinase; AD: Alzheimer’s disease; PD: Parkinson’s disease; MS: multiple sclerosis; MDD: major depressive disorder; IL-1: interleukin-1; TNF-α: tumor necrosis factor alpha; IL-6: interleukin-6; TBI: traumatic brain injury; NSAID: nonsteroidal anti-inflammatory drug; JAK/STAT: Janus kinase/signal transducer and activator of transcription; Aβ: amyloid-beta; MMP: matrix metalloproteinase; ASD: autism spectrum disorder; IFN-γ: interferon gamma; COX: cyclooxygenase; mAb: monoclonal antibody; RNAi: RNA interference; CRP: C-reactive protein; ASO: antisense oligonucleotide; ALS: amyotrophic lateral sclerosis; MSC: mesenchymal stem cell; CSF: cerebrospinal fluid; FUS: focused ultrasound; PLGA: poly(lactic-co-glycolic acid); sTREM2: soluble triggering receptor expressed on myeloid cells 2; GFAP: glial fibrillary acidic protein; NfL: neurofilament light chain; SIMOA: single-molecule array; EV: extracellular vesicle; PET: positron emission tomography; MRI: magnetic resonance imaging; TSPO: translocator protein 18 kDa; PBMC: peripheral blood mononuclear cell; LPS: lipopolysaccharide; SCFA: short-chain fatty acid.
